# Latent HIV in primary T lymphocytes is unresponsive to histone deacetylase inhibitors

**DOI:** 10.1186/1743-422X-8-400

**Published:** 2011-08-12

**Authors:** Gautam K Sahu, Miles W Cloyd

**Affiliations:** 1 Department of Microbiology and Immunology, University of Texas Medical Branch, 301 University Blvd, Galveston, TX-77555, USA; 2 Biotherapeutics Development Laboratory, Roger Williams Medical Center, 825 Chalkstone Avenue, NC-143, Providence, RI 02908, USA

## Abstract

Recently, there is considerable interest in the field of anti-HIV therapy to identify and develop chromatin-modifying histone deacetylase (HDAC) inhibitors that can effectively reactivate latent HIV in patients. The hope is that this would help eliminate cells harboring latent HIV and achieve an eventual cure of the virus. However, how effectively these drugs can stimulate latent HIVs in quiescent primary CD4 T cells, despite their relevant potencies demonstrated in cell line models of HIV latency, is not clear. Here, we show that the HDAC inhibitors valproic acid (VPA) and trichostatin A (TSA) are unable to reactivate HIV in latently infected primary CD4 T cells generated in the H80 co-culture system. This raises a concern that the drugs inhibiting HDAC function alone might not be sufficient for stimulating latent HIV in resting CD4 T cells in patients and not achieve any anticipated reduction in the pool of latent reservoirs.

## Background

The presence of resting memory CD4 T lymphocytes that harbor chromosomally integrated latent HIVs has been one of the major obstacles in eliminating HIV from patients using HAART [1,2]. Although the frequencies of these cells *in vivo *are very low (i.e., ~1-10 latently infected cells per million CD4 T cells), these highly stable cells serve as a life-long reservoir for HIV in infected individuals, despite long-term effective therapy [3]. To attain an eventual cure for HIV, if ever achievable, the elimination of these cells from patients is necessary, which remains extremely challenging and it is not clear how that can be achieved.

The mechanisms involved in HIV latency are multifactorial [4] and one of the underlying mechanisms found for the maintenance of latent HIV in transformed cell lines is the chromatin-mediated suppression of viral mRNA synthesis from the HIV LTR [5]. The treatment of latently infected cell lines with HDAC inhibitors, such as valproic acid (VPA) or trichostatin A (TSA), can increase the levels of histone acetylation [6,7], leading to changes in local chromatin organization at the latent HIV LTR [8-10]. This disruption of compact chromatin structure at the LTR causes higher levels of HIV transcription, resulting in the reactivation of latent HIV [10] and production of viral progeny in cell lines. Therefore, recent thrusts in the field are to identify potent HDAC inhibitors and use them clinically, because the assumption is that once HDAC inhibitors reactivate latent HIVs in resting CD4 T cells in patients, these cells will die because of virus-induced cytopathicity and/or HIV-specific cell-mediated immunity which is present. However, it is not entirely clear if these drugs can stimulate latent HIVs in primary CD4 T cells.

We have previously shown the formation of latent HIV at high-percentages in HIV-infected primary CD4 T cells *in vitro *using a feeder cell line, H80 [11], to keep the lymphocytes alive and healthy. Here, we have tested whether valproic acid or trichostatin A (both are HDAC inhibitors) can stimulate HIV in latently infected primary CD4 T cells generated in our H80 co-culture system.

## Results and Discussion

We generated latently HIV-infected primary T cells as previously described [11]. Briefly, we started with purified CD4 T cells from normal donors' blood. Cells were stimulated with cross-linked anti-CD3 on plastic dishes for 2 days. Then the cells were scrapped off and cultured in IL-2 media for 5 days, and were infected with a frozen stock of a CXCR4-dependent, low cytopathic HIV strain, MCK, at m.o.i. ~1. The infected cells were cultured for 2-3 weeks and then co-cultured on the H80 feeder cell line for 6 weeks as described previously [11]. Typically, ~5-10% of the CD4 cells present are latently infected by this time point.

**Figure 1:**
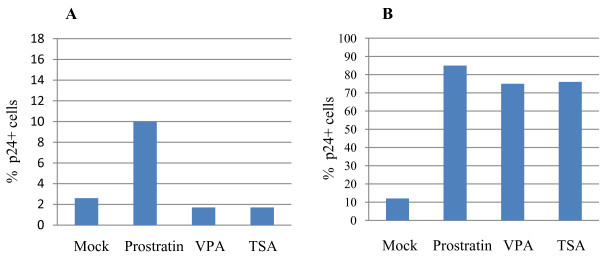
**Effect of prostratin and HDAC inhibitors on HIV-latency**. HIV-infected primary CD4 T cells cultured long-term on H80 (panel A) and ACH-2 cells (panel B) were treated with prostratin (500 nM), VPA (2 mM) and TSA (150 nM) for 18 hours and % increase in p24+ cells was determined by intracellular staining with HIV-core antibody (Coulter, USA) followed by flow cytometry as performed previously [11]. Mock indicates untreated control. One representative experiment out of 5 independent experiments with similar results is shown here.

The T lymyhocytes from co-culture were collected and examined for whether the HDAC inhibitors, VPA and TSA, could stimulate latent HIV in these primary cells, given that these compounds are known to reactivate latent HIVs in cell line models of HIV latency by disrupting suppressive chromatin structures at latent HIV LTRs. We treated ~0.5 × 10^6 ^cells with VPA (2 mM) and TSA (150 nM) separately for 18 hours. As a control for latent HIV stimulation, we also treated these cells with prostratin (500 nM), a non-tumor promoting phorbol ester and an activator of protein kinase C. In our previous study, we found we could reactivate latent HIV in primary CD4 T cells maintained in H80-cuture system using prostratin [11]. During these treatment periods, an HIV inhibitor, AZT, was also added at 0.13 μg/ml concentration to prevent HIV's spread in culture. Cells were then stained intracellularly with anti-p24 antibody (Coulter Inc., USA) and analyzed by flow cytometry. As can be seen in Figure 1A, there were about 2.5% of the T cells showing HIV-p24 positivity in mock (untreated) culture, and ~4-fold increase in the percentage of p24+ cells in culture upon prostratin-treatment because of reactivation of latent HIVs. However, when the cells were treated with VPA or TSA, the percentage of HIV-p24+ cells did not increase over mock (untreated) culture (Figure 1A), suggesting that VPA or TSA could not stimulate latent HIVs in these cells. In contrast, these drugs could effectively stimulate latent HIV in ACH-2 cells (Figure 1B), a transformed T cell line having most cells (~90%) latently infected with HIV.

**Figure 2:**
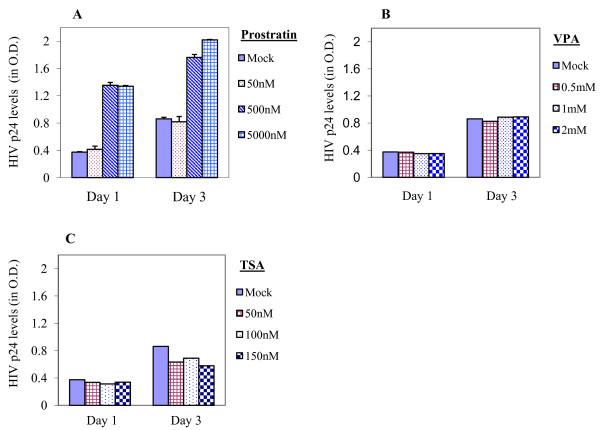
**Levels of HIV-p24 in culture supernatants post-stimulation of latent HIV**. About 0.5 × 10^6 ^of long-term cultured HIV-infected CD4 T cells that include latently-infected cells were treated with various concentrations of prostratin (A), VPA (B) and TSA (C) for 72 h. Culture supernatants were harvested 24 h and 72 h post-treatment and assayed for HIV-p24 levels by using p24-ELISA kit (Advanced Bioscience Laboratories, Inc., USA). The relative levels of HIV-p24 in various samples were expressed in O.D. values. Only prostratin, at 500 nM or higher concentrations but not VPA or TSA, could increase the levels of p24 in culture supernatants.

To further assess the stimulatory effects of these drugs on latent HIVs in primary T cells, we treated these cells co-cultured on H80 with various concentrations of prostratin, or VPA, or TSA, as indicated in Figure 2. Culture supernatants were harvested 24 h and 72 h after treatment and then assayed for HIV-p24 levels by using p24 antigen capture EIA (Advanced BioScience Laboratories, Inc., USA). Similar to the results obtained in flow cytometry, VPA or TSA treatment could not increase the levels of HIV-p24 in culture supernatants at any time points or concentrations tested over the levels observed in mock (untreated) cultures (Figure 2B and 2C), whereas prostratin-treatment (500 nM or higher) consistently led to relative increases in p24-levels in culture supernatants by > 2-fold compared to mock (untreated) control (Figure 2A). These data show that VPA or TSA-treatment could not cause stimulation of latent HIV in primary T-cells cultured long-term on H80 feeder cells.

**Figure 3:**
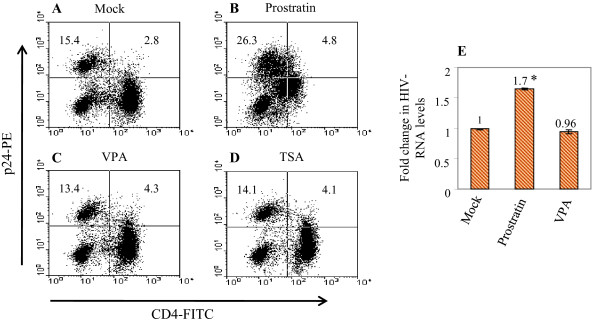
**VPA or TSA still remained ineffective on high percentages of latent HIVs in primary T cells**. Panel A, High percentages of latently infected cells were generated on H80 as described in the text and treated with with 500 nM prostratin (B), 2 mM VPA (C) and 150 nM TSA (D) for 18 hours. The percent increases in p24+ cells were determined by intracellular staining and flow cytometry. Untreated control (Mock) was shown in panel A. Panel E shows the relative changes in HIV-gag RNA levels after various treatments. The relative levels of intracellular HIV-gag RNAs were measured by real time quantitative RT-PCR (see text). Prostratin could induce the levels of HIV-gag RNA by 1.7-fold (p < 0.01) in primary cells, compared to untreated (mock) control, whereas VPA did not induce the level of gag-RNA in these cells. The numbers on top of each bar indicate fold change relative to untreated (mock) control. Results shown here are from triplicate samples in a representative experiment. Error bars indicate standard deviations. The statistically significant data (p < 0.01) determined by Student's t-test is indicated by an asterisk.

It can be argued that the low-percentages of latently infected T-cells present in H80 culture system (shown above) might be a confounding factor for assessing the responsiveness of latent HIVs to HDAC inhibitors. This might be because if a minor population among all latently infected T-cells generated in culture is actually susceptible to HDAC-inhibitor-mediated reactivation of latent HIVs, it could have been difficult to detect latent HIV's reactivation in our assays. Therefore, we increased the levels (percentages) of latently infected cells in culture as follows and then tested them: we infected normal CD4 T lymyhocytes with HIV-MCK 10-12 days after stimulation rather than at 5 days after stimulation as done above or previously [11]. Cells divide at ~5-fold lower rate at 10-12 days after stimulation than at peak proliferation phase on day 3 (data not shown). We found if primary CD4 lymphocytes are infected on day 10-12 after stimulation, much higher percentages of latently-infected, non-dividing cells are generated after co-culture on the feeder line, than obtained previously [11]. In one representative experiment out of 6 independent experiments, we found ~18% of the healthy, non-dividing T cells in long-term cultures were making very low levels of HIV chronically (Figure 3A). However, treatment with 500 nM prostratin could increase the percentages of HIV-p24+ cells to ~31% (Figure 3B), which was ~1.7-fold increase over untreated controls (see Table 1). Therefore on average ~15% of cells were latently infected with HIV in these cultures (Table 1). Of note, prior to these flow cytometric analyses, these cells were cultured for 5 days in the presence of AZT at 0.13 μg/ml concentration to prevent the spread of HIV in culture. Then we treated these cells with VPA or TSA for 18 hours and stained intracellularly with HIV-core antibody (Coulter Inc. USA) to compare relative percentages of HIV-producing cells with or without stimulation. We found in these cultures, also, that VPA or TSA could not increase the percentages of HIV-p24+ cells (Figure 3, compare panel A with C and D, also see Table 1), further showing that these HDAC inhibitors were not effective in reactivating latent HIVs in primary quiescent T cells generated on H80 feeder cells.

**Table 1:** Changes in the percentages of p24+ cells in HIV-infected quiescent cultures upon treatment with various chemicals

Chemicals used to stimulate latent HIVs in primary T cells	Average % p24+ cells determined by flow cytometry	Fold-change in average % p24+ cells in treated vs. untreated groups
None (untreated)	18.9 ± 4.3	

Prostratin (500 nM)	33.7 ± 4.2	1.78

VPA (2 mM)	18.0 ± 6.2	0.95

TSA (150 nM)	17.1 ± 3.0	0.90

The data were compiled from six independent experiments. ± numbers represent standard deviations.

To measure the relative levels of HIV RNA in treated versus untreated HIV-infected primary T cells, we carried out real-time quantitative RT-PCR for HIV-*gag *sequences as follows: total RNA isolated was quantified using a Nanodrop Spectrophotometer (Nanodrop Technologies) and qualified by analysis on an RNA Nanochip using the Agilent 2100 Bioanalyzer (Agilent Technologies). Synthesis of cDNA was performed with 1 μg of total RNA in a 20 μl reaction volume for 30 min at 48°C using the reagents in the Taqman Reverse Transcription Reagents Kit (Applied Biosystems). Real-time quantitative PCR amplifications were performed in triplicates using 2 μl of cDNA in a total volume of 25 μl using TaqMan MGB probe with the TaqMan Universal PCR Master Mix (Applied Biosystems). The final concentration of the probe was 250 nM and of the primers were 900 nM. Based on the HIV strain (HIV-MCK) used in our experiments, the designed probe sequence was 5'-ACCCCACAAGATTTAAA-3', and the primer sequences were: forward, 5'-AATACCCATGTTTTCAGCATTATCAGA-3' and reverse, 5'-TGATGTCCCCCCACTGT GTT-3'. Relative quantitative RT-PCR assays were performed with 18S RNA as a normalizer. All PCR assays were run in the ABI Prism 7000 Sequence Detection System and the conditions were as follows: initial incubation (50°C for 2 min) and denaturation (95°C for 10 min) steps were followed by 40 cycles of amplification (each cycle: 95°C for 15 sec and 60°C for 1 min). The analysis showed that prostratin-treatment could increase HIV-RNA levels by 1.7-fold (p < 0.01) as shown in Figure 3E, with a concomitant increase in the percentages of HIV-p24+ cells by ~1.8-fold (see Table 1). In contrast, HIV-RNA levels in latently-infected primary T cells remained unchanged after VPA-treatment (Figure 3E) as did the percentages of HIV-p24 positive cells (Table 1). These data presented here demonstrate that latent HIVs in primary quiescent CD4 T-lymphocytes are not responsive to the HDAC-inhibitors VPA or TSA.

One of the recent thrusts in the field of HIV treatment focuses on identifying potent HDAC-inhibitors for use clinically as an adjunct therapy to HAART. This endeavor stems mainly from previous observation by Lehrman *et. al*. [12] who showed the reduction in the pool of latently infected resting CD4 T cells by ~75% in 3 of 4 patients when treated with VPA in combination with suppressive HAART. This study, although not definitive, suggested the possibility that the current antiviral regimens plus additional drugs targeting latent reservoirs may attain cures of HIV in chronically infected patients. Although subsequent studies by others did not find similar effects of VPA on latent reservoir in patients [13,14], these studies opened up new avenues for antiviral research aiming to break HIV latency in resting CD4 T cells in order to achieve depletion of latently infected cells in patients on HAART. However, our data demonstrate that VPA or TSA are unable to drive the expression of virus from its latent state in primary resting CD4 T cells, in contrast to their well-documented effectiveness in transformed, actively dividing cell line models of HIV latency. Our data are also in agreement with the previous observation by Brooks *et. al*. [15] who did not observe reactivation of latent HIV in primary T cells obtained from the SCID-hu (Thy/Liv) mouse model of HIV-latency.

Although we do not know whether VPA or TSA can remodel chromatin organization at HIV LTRs in latently infected primary T lymphocytes generated in our culture system, the requirements for reactivating latent HIVs appear multifactorial [4]. For example, resting T cells possess limiting amounts of various transcription factors, such as activated nuclear NF-kB, Cyclin T1 and Cdk9 required for productive transcription to occur from the HIV LTR [16,17]. There is no evidence indicating that VPA or TSA-treatment can induce the expression of these factors in resting primary CD4 T cells or activate these cells. As a cautionary note, our data predict that the use of HDAC-inhibitors as adjunct therapy in the "shock and kill" approach for depleting latent reservoirs in patients would not be fruitful, unless simultaneous activation or induction of expression of cellular factors (such as NF-kB, CycT1, Cdk9 etc.) essential for high-level HIV gene expression is achieved in resting CD4 T cells.

It is worthwhile to mention that during the last decade, latently infected transformed T cell clones have been extensively used in the context of mechanistic studies of the establishment and the maintenance of HIV latency and its reactivation from the latent state. However, many of these clones were isolated by screening and selecting the clones that were responsive to TNF-α-mediated stimulation of latent HIV constructs [18]. Recently Tyagi *et. al*. [19] have used the H80 system to generate latently infected primary T cells at high-percentages *in vitro *using pseudotyped HIV (negative for its own envelope), and found TNF-α could not reactivate latent HIVs in these cells. This is because of restricted cellular levels of P-TEFb (CDK-9, CycT1) in spite of nuclear NF-kB activation by TNF-α signaling in these cells [19]. Although we generated latently infected primary T-cells in the H80 system using infectious Env+ HIV at lower percentages than what Tyagi et. al. [19] did; we, likewise, did not observe activation of latent HIV in these cells upon TNF-α treatment (data not shown). Altogether, our and others data obtained from HIV-infected primary T cells cultured on H80 strongly advocate for studying HIV latency in non-dividing quiescent normal CD4 T cells, rather than in transformed, actively dividing cell lines.

## List of abbreviations

HIV: Human Immunodeficiency virus-1; VPA: Valproic acid; TSA: Trichostatin A; HDAC: Histone deacetylase; HAART: Highly active anti-retroviral therapy; LTR: Long-terminal repeat; SCID-hu (Thy/Liv): Severe combined immunodeficiency-human (thymus/liver).

## Competing interests

The authors declare that they have no competing interests.

## Authors' contributions

GKS conceived of the study, participated in the design, carried out most of the assays, analyzed the data and drafted the manuscript. MWC participated in the design and analyses. Both authors read and approved the final manuscript.
